# Role of GOLPH3 and TPX2 in Neuroblastoma DNA Damage Response and Cell Resistance to Chemotherapy

**DOI:** 10.3390/ijms20194764

**Published:** 2019-09-25

**Authors:** Marzia Ognibene, Marina Podestà, Alberto Garaventa, Annalisa Pezzolo

**Affiliations:** 1 Laboratorio Cellule Staminali Post Natali e Terapie Cellulari, IRCCS Giannina Gaslini, 16147 Genova, Italy; marinapodesta@gaslini.org; 2 Divisione di Oncologia, IRCCS Giannina Gaslini, 16147 Genova, Italy; albertogaraventa@gaslini.org

**Keywords:** Neuroblastoma, DNA-damage, GOLPH3, resistance to drugs, TPX2, autophagy

## Abstract

Neuroblastoma (NB) is an aggressive, relapse-prone infancy tumor of the sympathetic nervous system and is the leading cause of death among preschool age diseases, so the search for novel therapeutic targets is crucial. Golgi phosphoprotein 3 (GOLPH3) has been reported to be involved in the development, and in the DNA damage response, of various human cancers. Golgi dispersal is a common feature of DNA damage response in mammalian cells. Understanding how cells react to DNA damage is essential in order to recognize the systems used to escape from elimination. We induced DNA damage in two human neuroblastoma cell lines by curcumin. The exposure of neuroblastoma cells to curcumin induced: (a) up-regulation of GOLPH3^+^ cells; (b) augmentation of double-strand breaks; (c) Golgi fragmentation and dispersal throughout the cytoplasm; (d) increase of apoptosis and autophagy; (e) increased expression of TPX2 oncoprotein, able to repair DNA damage. Primary neuroblastoma samples analysis confirmed these observations. Our findings suggest that GOLPH3 expression levels may represent a clinical marker of neuroblastoma patients’ responsiveness to DNA damaging therapies—and of possible resistance to them. Novel molecules able to interfere with GOLPH3 and TPX2 pathways may have therapeutic benefits when used in combination with standard DNA damaging therapeutic agents in neuroblastoma

## 1. Introduction

Neuroblastoma (NB), the most common extra-cranial solid cancer in children, originates from the precursors of the sympathetic nervous system [1]. The clinical courses of the disease are deeply heterogeneous: half of the tumors regresses spontaneously or after short therapies, while the other half is lethal in the majority of patients despite multiple stronger treatments. In the current treatment stratification system, the clinical and biological prognostic variables such as age at diagnosis, stage of disease, amplification of the proto-oncogene *MYCN*, and segmental chromosome copy number alterations are central for NB risk estimation [2,3,4,5,6]. However, a number of patients are deceased due to the resistance to chemotherapeutic drugs. Therefore, to prolong the survival of patients affected by NB, it is crucial to determine the mechanism underlying the occurrence of drug resistance and find an effective treatment method to increase drug sensitivity. Multidrug resistance is the major cause for treatment failure in cancer including NB [7,8,9,10,11,12,13,14,15]. NB cells can resist drugs taking advantage of apoptosis deregulation [9,10], the presence of cancer stem cells [11,12], alterations of drug targets [13], augmented capacity of DNA repair [14], and faster drug effluence [15]. Tumor cells can further enhance their drug resistance by exploiting autophagy mechanisms, a lysosomal degradation pathway responsible for the withdrawal of aged organelles and proteins [16]. Previous studies have indicated that therapeutic agents can induce autophagy, so facilitating cancer cell survival during drug-induced metabolic stress [16].

Golgi phosphoprotein 3 (GOLPH3) is a well conserved membrane protein located in the Golgi complex and is able to move between the Golgi and endosomal system, so that it is involved in Golgi trafficking and morphology preservation [17,18,19]. The GOLPH3 pathway is indispensable for vesicular trafficking from the Golgi to the plasma membrane [20]. GOLPH3 binds to MYO18A (Myosin XVIIIA) and, through this protein, it links Golgi to the actin cytoskeleton [21,22]. A side effect of GOLPH3 dependent trafficking is to create the extended ribbon shape of the Golgi. Disturbance of the pathway results in changes to both Golgi morphology and secretion, with functional consequences for the cell [17]. Field and colleagues reported that DNA dependent protein kinase complex, also known as DNA-PK, can phosphorylate GOLPH3 in response to DNA damage, and consequently, GOLPH3 increases its interaction with MYO18A and its tensile force on the Golgi, leading to Golgi vesiculation and dispersal throughout the cytoplasm [23]. The DNA-PK/GOLPH3/MYO18A pathway is necessary for cell survival after DNA damage, and therefore GOLPH3 over-expression gives resistance to elimination induced by DNA damaging agents [24,25,26,27]. Errors in DNA damage response contribute to genome instability, a hallmark of cancer, which, in turn, leads to tumor progression [28]. Previous research has demonstrated that *GOLPH3* is a proto-oncogene [29,30,31,32,33,34]. The over-expression of GOLPH3 confers survival benefit to cancer cells, suggesting that Golgi fragmentation is necessary for cell survival. Thus, Golgi fragmentation may contribute to tumor development and maintenance, giving rise to an endless loop [35,36]. Understanding the cellular response to DNA damage is crucial for discerning the mechanism by which many chemotherapeutic agents kill tumor cells and the mechanism of escape from elimination [37].

Several studies have paid attention to the relationship between TPX2 and DNA damage response [38,39]. TPX2 is a protein involved in spindle apparatus assembly and specifically associated to cell microtubules [38]. The *TPX2* gene is part of the signature of chromosomal instability from specific genes whose expression was consistently correlated with clinical outcome in multiple human cancers [40,41], and it has been identified as a driving oncogene in different kinds of neoplasm [42,43,44,45,46,47]. To the best of our knowledge, GOLPH3 associated resistance to chemotherapy and its underlying mechanism in human NB have not been previously reported. In this study, we show the linkage between DNA damage and GOLPH3 expression in NB, and how this can cause cell resistance even through higher levels of *TPX2* oncogene expression. 

## 2. Results

### 2.1. Cytotoxicity Induced by Curcumin

**Figure 1:**
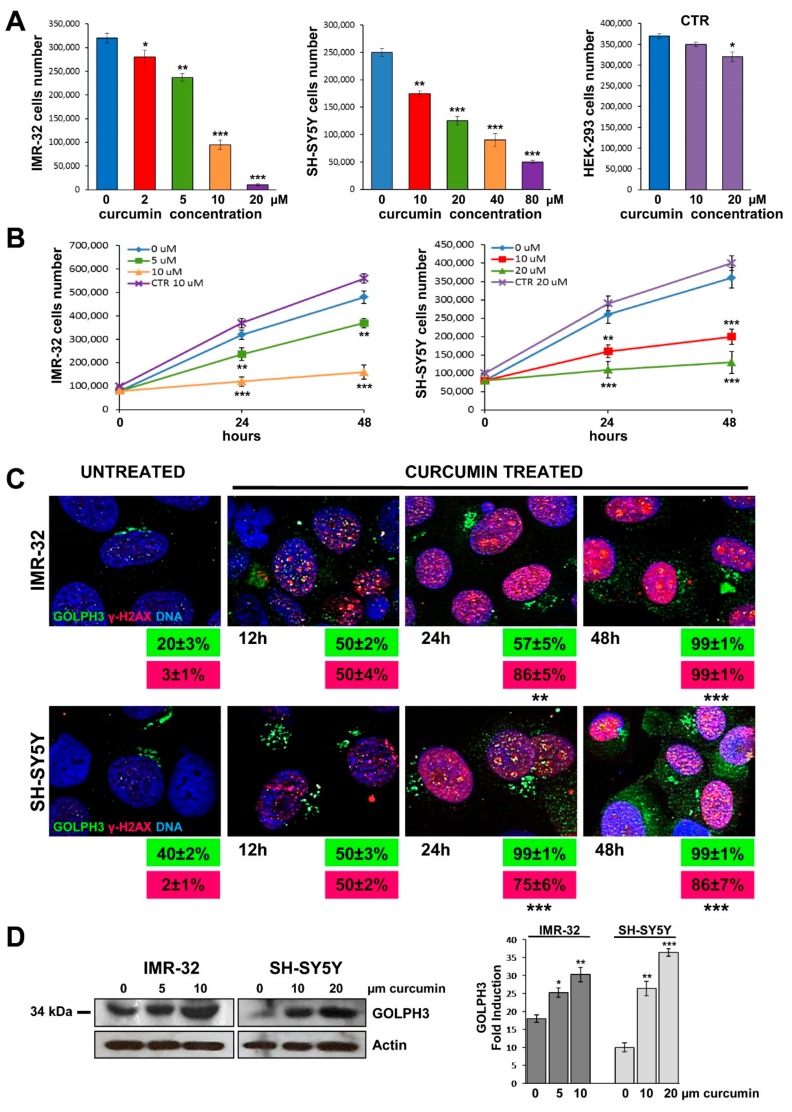
Curcumin provoked DNA damage in neuroblastoma cells and up-regulation of GOLPH3 with Golgi dispersal. (**A**) IMR-32, SH-SY5Y and non-tumorigenic control HEK-293 (CTR) cell lines were cultured in presence of various concentrations of curcumin for 24 h. (**B**) Cells were cultured with two curcumin concentrations for 24 and 48 h. At each harvest point, cells were trypsinized and counted in Trypan blue. Untreated cells (curcumin 0 µM) were cultured with 0.1% DMSO. Non-tumorigenic control HEK-293 cells (CTR) were cultured with the highest curcumin concentrations used for each NB cell line. Data are representative of three independent experiments ± SD. (**C**) Immunofluorescence analysis of IMR-32 and SH-SY5Y cells cultured with 10 or 20 µM curcumin respectively for 12, 24 and 48 h using anti-GOLPH3 (green) and anti-γH2AX (red). Cells were counterstained with DAPI to visualize nuclei (blue). Untreated cells were cultured with 0.1% DMSO. (Magnification 40×). In green and red boxes are reported the percentages of GOLPH3 and γH2AX positive cells respectively. Data are representative of three independent experiments ± SD. (**D**) IMR-32 and SH-SY5Y cells were cultured in presence of two concentrations of curcumin for 48 h, lysed, subjected to Western blot analysis and probed with anti-GOLPH3 antibody. Controls (curcumin 0 µM) were treated with 0.1% DMSO. Protein level was quantified by densitometry, normalized to the content of the loading control protein (actin) and visualized by histogram. Data are representative of three independent experiments ± SD (* *p* < 0.05; ** *p* < 0.01; *** *p* < 0.001)).

We induced DNA damage by treating with curcumin two NB cell lines IMR-32, *MYCN* amplified, and SH-SY5Y, *MYCN* single copy with strong c-MYC protein expression, in a dose dependent manner for 24 h at 37 °C. The cell viability was measured vs untreated cells as an indicator for cytotoxicity of the compound. Growth rate was sensibly lower with 5–10 µM of curcumin for IMR-32 cells, and with 10–20 µM of curcumin for SH-SY5Y cells. As a non-tumorigenic control cell line, we chose human embryonic kidney HEK-293 cells (see Materials and Methods), that were cultured for 24 h with the highest curcumin concentrations used for each NB cell line, with no effects on their viability (Figure 1A).

Next, we tested the effects of curcumin treatment on cells proliferation after 24 and 48 h, using the two most significant concentrations of the compound for each cell line. We observed a clear growth decline of treated NB cells vs. control cells after 48 h of incubation with 10 µM of curcumin for IMR-32 cells, or with 20 µM for SH-SY5Y cells (Figure 1B). Then we performed all the following experiments treating IMR-32 and SH-SY5Y cells for 48 h with curcumin 10 and 20 µM respectively.

### 2.2. Curcumin Induces DNA Damage in Neuroblastoma Cells

For further confirmation that curcumin reduced the percentage of total viable NB cells through the induction of DNA damage, we performed an analysis of γH2AX expression by immunofluorescence analysis on NB cells treated with curcumin. The histone H2AX is a highly conserved globular protein that is structurally involved in packaging and organizing DNA into chromatin. Upon phosphorylation of serine-139 near the C-terminus end, γH2AX fast localizes to DNA double-strand breaks [48]. As such, γH2AX is generally considered as a marker of DNA damage [49]. An example of γH2AX foci induced by treatment with curcumin is shown in Figure 1C. The results demonstrated a significant increase in number of DNA damage foci into the nucleus of curcumin treated NB cells compared with untreated cells. The percentage of γH2AX^+^ nuclei, that was almost absent in untreated cells, increased sensibly after 12 and 24 h and reached 86% or 100% after 48 h of curcumin treatment (*p* < 0.001) (Figure 1C). The non-tumorigenic control HEK-293 cell line cultured with curcumin 20 µM for 48 h displayed no double-strand breaks, so confirming that curcumin effect inducing DNA damage is specific for cancer cell lines (Supplementary Figure S1).

### 2.3. Curcumin Promotes GOLPH3 Up-Regulation and Dispersal of Golgi in Neuroblastoma Cells

We examined whether DNA damage affects Golgi morphology in NB cells. For this purpose we performed immunofluorescence analysis on NB cells treated with curcumin 10 µM for IMR-32 or 20 µM for SH-SY5Y for 12, 24 and 48 h. GOLPH3 expression increased over time together with DNA damage, and we observed an extended ribbon shape of the Golgi in association with augmentation of double-strand breaks γH2AX^+^(Figure 1C). Exposure to each treatment of curcumin caused Golgi morphology to change drastically from the usual perinuclear ribbon to punctuate fragments dispersed throughout the cytoplasm. The percentage of GOLPH3^+^ NB cells treated with curcumin in independent experiments increased, starting from 20% or 40% in untreated cells, to 100% after 48 h (*p* < 0.001) (Figure 1C).

Moreover, NB cells cultured in different concentrations of curcumin for 48 h were lysed and subjected to Western blot. GOLPH3 protein level resulted augmented after treatment with curcumin, especially when used more concentrated (Figure 1D).

We treated IMR-32 and SH-SY5Y cells with other three chemotherapy compounds, chosen for their known anti-proliferative actions on NB cells, in order to test their effect on inducing DNA damage and Golgi fragmentation: (a) Rapamycin, an inducer of autophagy by inhibiting mTOR, the Ser/Thr protein kinase that regulates cell growth and metabolism in response to environmental cues [50] (b) Bortezomib, a chemoterapic agent belonging to proteasome inhibitors family, with stabilizing effect on molecules able to block cell survival and cell cycle progression [51] and (c) Colchicine, a microtubule disrupting agent that induces apoptosis in NB cells [52]. The immunofluorescence analysis on cells treated with different concentrations of these compounds for 48 h showed a lower γH2AX and GOLPH3 positivity in comparison with the results obtained with curcumin, in terms of both number of positive cells and staining intensity (Supplementary Figure S2 and Appendix A). On this basis, we decided to carry on the experiments with curcumin, which causes evident DNA damage and Golgi fragmentation, and less cytotoxicity to NB cells.

### 2.4. Curcumin Reduces Activation of MAP-Kinases and Induces Apoptosis and Autophagy in Neuroblastoma Cells

**Figure 2:**
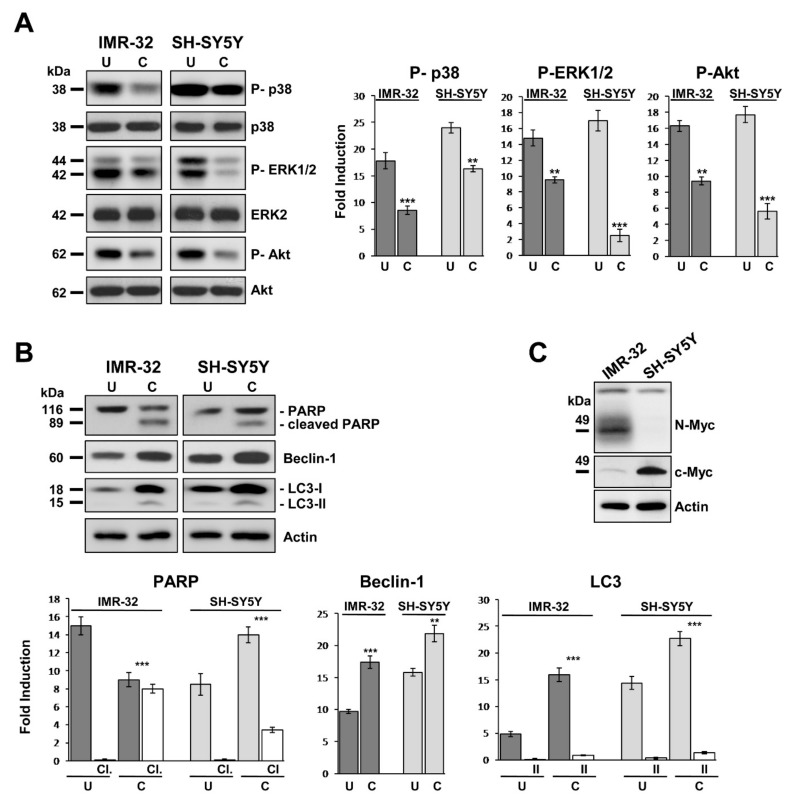
Curcumin reduces activation of MAP-kinases and induces apoptosis and autophagy in neuroblastoma cells. (**A**) Protein lysates from IMR-32 and SH-SY5Y cells cultured in 10 or 20 µM curcumin respectively for 48 h (C = curcumin) were collected and subjected to Western blot analysis with anti-phospho-p38, anti-phospho-ERK 1/2, and anti-phospho-Akt antibodies. (**B**) The same protein lysates were blotted again and probed with anti-PARP for apoptosis (cl = cleaved PARP), and anti-Beclin-1 or anti-LC3 antibodies for autophagy (II = LC3-II). Untreated cells (U) were cultured with 0.1% DMSO. Protein levels were quantified by densitometry, normalized to the content of the loading control protein (each total protein or actin) and visualized by histograms. Data are representative of three independent experiments ± SD. (**C**) MYCN and c-MYC expression in IMR-32 and SH-SY5Y cell lines displayed by Western blot (** *p* < 0.01; *** *p* < 0.001).

Next, we considered how curcumin inhibits NB cell proliferation by inducing DNA damage and Golgi dispersal. The reduced growth rate observed in Figure 1B was associated with decreased of phosphorylation of mitogen-activated protein kinases (MAPKs) (Figure 2A). Cells cultured in presence of curcumin for 48 h showed that p38 MAPK (p38) was 50% less activated than control for IMR-32, and 30% less for SH-SY5Y. In the same way, extracellular signal regulated kinase 1 and 2 (ERK1/2) resulted 36% less phosphorylated in treated IMR-32 cells and 85% less in treated SH-SY5Y than their respective controls (Figure 2A).

We evaluated the activation state of RAC-alpha serine-threonine-protein kinase (AKT), the major downstream effector of phosphoinositide 3-kinase (PI3K), to investigate the connection between curcumin effects and apoptosis in NB cells. The phosphorylation of Akt prevents apoptosis and induces cell proliferation and transformation. Treatment with curcumin caused in IMR-32 and SH-SY5Y cells a decrease of P-Akt by approximately 40% and 68% respectively in comparison to controls (Figure 2A). Apoptosis induction is proven by the DNA repair enzyme Poly ADP-Ribose Polymerase (PARP) cleavage, which was detectable exclusively in curcumin treated NB cells (Figure 2B). In addition, cells reaction to curcumin triggered autophagy, as evidenced by up-regulation of Beclin-1 and autophagic-vesicle-form LC3-II (Figure 2B). IMR-32 cells are *MYCN* amplified while SH-SY5Y cells are *MYCN* single copy with high c-MYC protein expression (Figure 2C).

**Figure 3:**
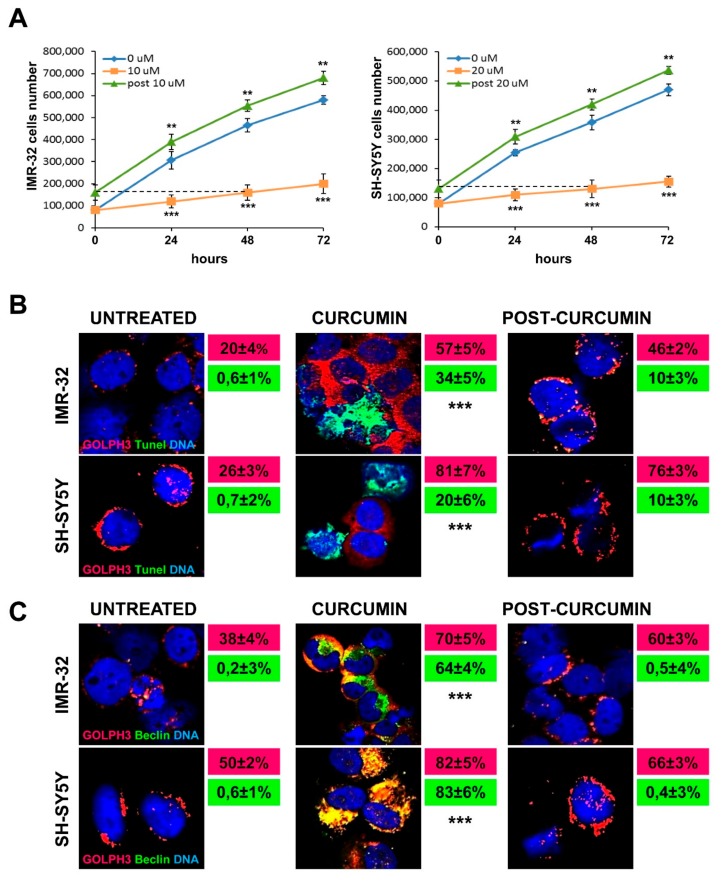
Golgi fragmentation is related to autophagy, not to apoptosis in neuroblastoma cells. (**A**) Comparison among the daily growth rate of untreated cells, of cells treated with curcumin and of cells recovering from 48 h of curcumin treatment, during three days of observation. For the recovery experiment (indicated as post 10 µM and post 20 µM) cells were incubated for 48 h with curcumin 10 or 20 µM and then washed and cultured in drug-free medium for 24, 48 and 72 h. The dashed line indicates the starting point of the recovery experiment observation, corresponding to the 48 h of curcumin treatment. At each harvest point, cells were trypsinized and counted in Trypan blue. Controls (curcumin 0 µM) were treated with 0.1% DMSO. Data are representative of three independent experiments ± SD. (**B**) Immunofluorescence analysis of IMR-32 and SH-SY5Y cells cultured with 10 or 20 µM curcumin respectively for 48 h and after 48 h post-Curcumin depletion, using anti-GOLPH3 (red) and TUNEL staining (green) to reveal chromatin condensation. In red and green boxes are reported the percentages of GOLPH3 and tunel positive cells respectively. (**C**) Immunofluorescence analysis of IMR-32 and SH-SY5Y cells using anti-GOLPH3 (red) and anti-Beclin-1 (green). In red and green boxes are reported the percentages of GOLPH3 and Beclin-1 positive cells respectively. Cells were counterstained with DAPI to visualize nuclei (blue). Untreated cells were cultured with 0.1% DMSO. (Magnification 40×). Data are representative of three independent experiments ± SD (** *p* < 0.01; *** *p* < 0.001).

Subsequently, we exposed NB cells to curcumin for 48 h to evaluate cells recovery degree after compound removal. To address this issue, we changed the medium with fresh complete DMEM and we counted viable cells after 24, 48 and 72 h. Cells growth, that was drastically fallen with curcumin treatment, started rapidly rising again already after 24 h from compound depletion for both IMR-32 and SH-SY5Y cells, and remaining constant over time. The comparison between the daily growth rate of untreated cells and the cells recovered from 48 h of curcumin treatment, indicated that the latter grew likewise the former, or even increasing their number every day of 10 cells more than controls, on average. On the contrary, during curcumin treatment NB cells increased themselves of 120–100 units less than controls on average every day. These experiments proved that curcumin affected proliferation without irreversibly compromising NB cells faculties, allowing cells to restore or actually to improve their growth rate (Figure 3A).

Immunofluorescence experiments revealed that in both IMR-32 and SH-SY5Y cells apoptosis induced by curcumin occurred not in concurrence with Golgi dispersal (Figure 3B). On the contrary, autophagy induced by curcumin treatment, showed by Beclin-1 expression, appeared co-localized with higher GOLPH3 expression (Figure 3C). Curcumin depletion after 48 h led to: (a) TUNEL-positive staining dramatically decreased; (b) slight downregulation of GOLPH3 expression; (c) rapid condensation of the Golgi apparatus around the nuclei; (d) inhibition of autophagy (Figure 3B,C).

### 2.5. GOLPH3 Up-Regulation is Related With Higher Expression of MYO18A and TPX2 in Neuroblastoma Cells

**Figure 4:**
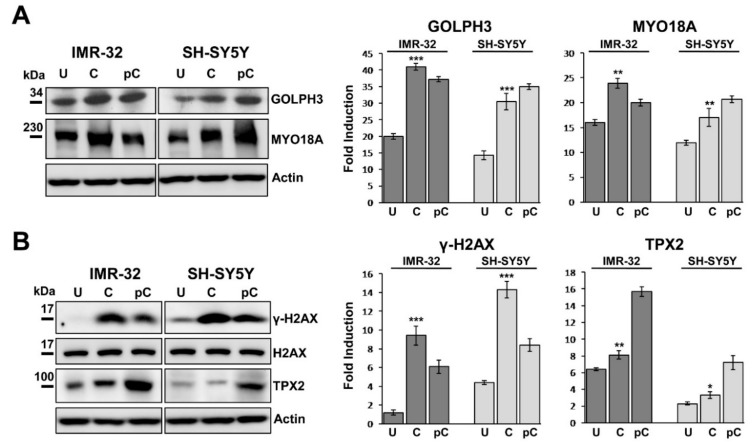
Curcumin treatment induces MYO18A up-regulation and TPX2 activation in neuroblastoma cells. (**A**) Protein lysates from IMR-32 and SH-SY5Y cells cultured in 10 or 20 µM curcumin respectively for 48 h (C = curcumin) and lysates from the same cells collected 48 h after curcumin depletion (pC = post-curcumin), were subjected to Western blot analysis with anti-GOLPH3 and anti-MYO18A antibodies. (**B**) The same lysates were subjected to Western blot analysis with anti-γH2AX and anti-TPX2 antibody. Untreated cells (U) were cultured with 0.1% DMSO. Protein levels were quantified by densitometry, normalized to the content of the loading control protein (total H2AX or actin) and visualized by histograms. Data are representative of three independent experiments ± SD (* *p* < 0.05; ** *p* < 0.01; *** *p* < 0.001).

The phosphorylation of GOLPH3 results in augmented interaction between GOLPH3 and MYO18A, a novel putative cancer driver, well known to be involved in the Golgi pathway [21,22,23]. We next examined by western blotting if the augmented expression of GOLPH3 in curcumin treated NB cells could be associated with MYO18A expression and Golgi dispersal. IMR-32 and SH-SY5Y cultured in presence of curcumin for 48 h indicated that MYO18A was clearly more expressed than in control cells, following GOLPH3 trend (Figure 4A). After 48 h since curcumin was removed from the growth medium, remarkable GOLPH3 and MYO18A expression was still maintained in both NB cell lines (Figure 4A).

The expression of γH2AX in cells cultured for 48 h with curcumin was extremely high, while it was almost undetectable in the untreated cells, as already observed in immunofluorescence (Figure 4B). After 48 h from curcumin depletion, γH2AX expression was halved (Figure 4B). Since TPX2 plays a principal function in the DNA damage response pathway and co-localizes with γ-H2AX at chromosomal double strand breakage sites [38,39], we analyzed the expression of TPX2 on the same cells and we found an opposite trend: TPX2 was activated by curcumin treatment but it resulted evidently up-regulated when the cells recovered from that treatment started growing again, indicating a proliferation comparable to control cells (Figure 3A, 4B). TPX2, by inducing DNA repair capacity, may help cells eliminating DNA breaks, so diminishing γH2AX level and increasing proliferation because of a restored cell physiology [53]. Augmented proliferation may be an effect of the autophagic reaction too, occurring in the resistant cells after curcumin treatment. We found different basal TPX2 protein expression in the two NB cell lines analyzed, which may be correlated to the fact that IMR32 cells are *MYCN* amplified and SH-SY5Y cells are *MYCN* single copy with strong c-MYC protein expression (Figure 2C, 4B).

### 2.6. Chemotherapy Increases GOLPH3, γH2AX and TPX2 Expression in Neuroblastoma Cells

**Figure 5:**
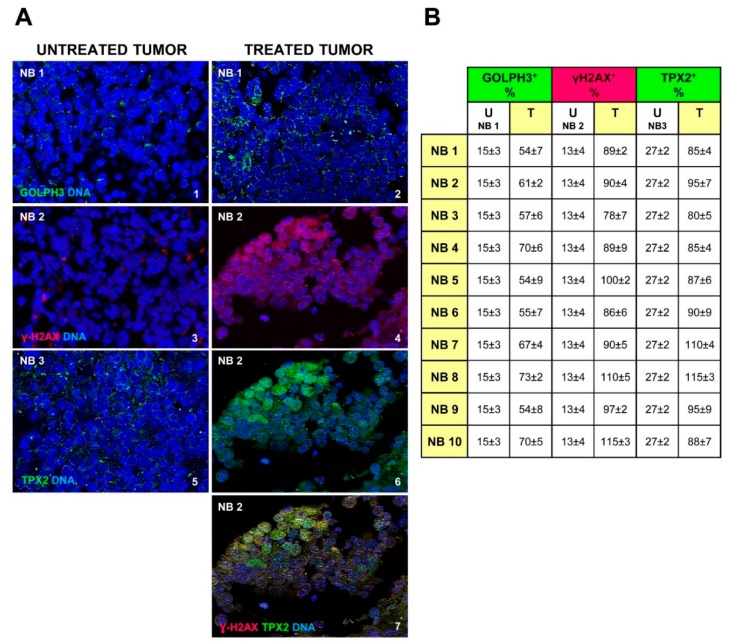
Golgi dispersal, DNA damage and expression of TPX2 oncogene are effects of chemotherapy in neuroblastoma (NB) patients. (**A**) Representative images of immunofluorescence analysis of NB tumor samples before (NB1, NB2, NB3) (1,3,5) and after chemotherapy (NB1, NB2) (2,4,6), by anti-GOLPH3 antibody (green) (1,2), anti-γH2AX antibody (red) (3,4) and anti-TPX2 antibody (green) (5,6). Image 7 comes from image 4 merged with image 6, to show the co-localized expression of the two proteins. Cells nuclei are counterstained with DAPI (blue). (Magnification 40×). (**B**) In the table are reported the percentages of GOLPH3, γH2AX and TPX2 positive cells observed in the 10 NB chemotherapy treated samples (NB1 to NB10), compared to a not treated NB specimen (NB1, NB2 and NB3) (U = untreated tumor; T = chemotherapy treated tumor). Data are representative of three independent observations ± SD.

We stained by immunofluorescence 10 NB human tissue sections coming from post-chemotherapy surgical resection. The clinical characteristics of the examined NB tumors are detailed in Table 1. Anti-GOLPH3 staining showed an increase of this molecule expression, and dispersal of the Golgi in the cytoplasm (GOLPH3^+^ cells 62 ± 3% on average vs. 15 ± 3% in the untreated tumor; *p* < 0.001), denoting an effect very similar to that observed in curcumin treated NB cells (Figure 5A(1,2) and Figure 5B). In the same manner, we showed higher positivity of γH2AX in cell nuclei of post-chemotherapy tumors (Figure 5A(3,4) and Figure 5B) (γH2AX^+^ cells 94 ± 2% on average vs. 13 ± 4% in the untreated tumor; *p* < 0.001) indicating augmented DNA damage. All samples obtained from patients with high-risk disease after rapid COJEC chemotherapy treatment, exhibit TPX2 expression increased too (TPX2^+^ cells 93 ± 3% on average vs. 27 ± 2% in the untreated tumor; *p* < 0.001) (Figure 5A(5,6) and Figure 5B), suggesting that TPX2 protein level is closely connected to DNA damage response in NB, and can confer upon the resistant cells the capability to rescue and to reproduce themselves again (Figure 5A(7)).

The genomic profile analysis of DNA extracted from these NB tumors obtained by pre-chemotherapy surgical resection, performed with array-CGH, disclosed multiple segmental chromosome gains or losses, index of disease poor outcome [54], in all specimens. Partial chromosome losses were most often detected for chromosomes 1p and 11q, while partial chromosome gain for 17q, and *MYCN* amplification was observed in eight samples out of 10 (Table 1). 

### 2.7. High TPX2 Gene Expression is Significantly Associated With Poor Prognosis in Neuroblastoma Patients

**Figure 6:**
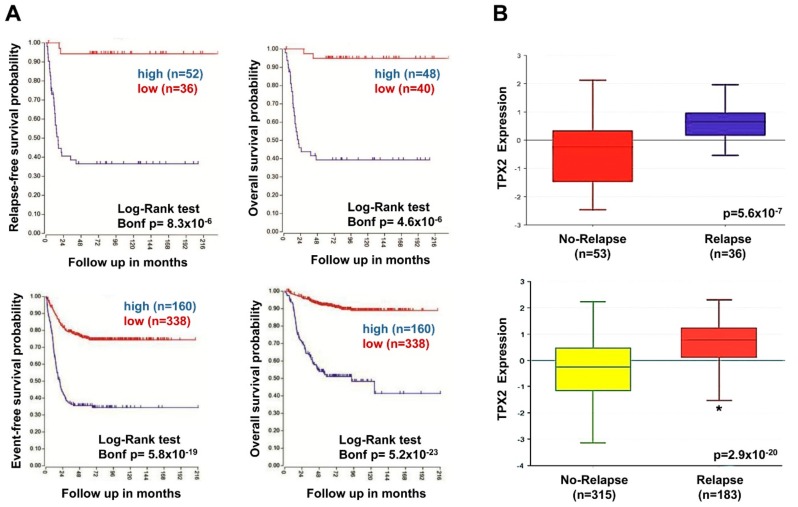
Neuroblastoma patient outcomes based on *TPX2* expression. (**A**) Using the neuroblastoma Versteeg (top) and SEQC (bottom) patient datasets in the R2 Genomics Analysis and Visualization Platform (http://r2.amc.nl), patients were divided into high (blue) and low (red) *TPX2* gene expression groups and survival curves were generated. Relapse-free survival (top left), event-free survival (bottom left) and overall survival (right) curves are shown with patient numbers in parentheses. (**B**) Relative *TPX2* expression levels were plotted for patients without and with relapse from the Versteeg (top) and SEQC (bottom) patient data sets, with patient numbers shown in parentheses (* *p* < 0.05).

We analyzed the data from microarray results obtained for NB tumor samples from R2 Genomics Analysis and Visualization Platform (http://r2.amc.nl), using the SEQC-498, and Versteeg databases that included complete information about clinical data. High expression of the *TPX2* gene was significantly linked with reduced event-free and overall survival in two different NB patient groups (Figure 6A). Furthermore, *TPX2* gene expression was significantly higher among relapsed versus not relapsed patients (Figure 6B).

**Figure 7:**
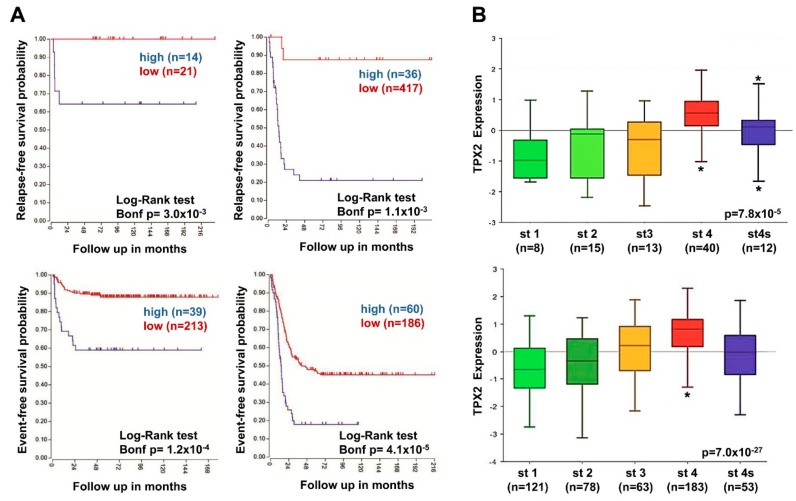
Neuroblastoma patient outcomes based on *TPX2* expression and disease stage. (**A**) Using the neuroblastoma Versteeg (top) and SEQC (bottom) patient datasets, survival curves were generated for patients with stage 1, 2, and 4S tumors (left) and with stage 3 and 4 tumors (right). Relapse-free survival (top) and event-free survival (bottom) curves are shown, with patient numbers in parentheses. (**B**) Relative *TPX2* expression levels from the Versteeg (top) and SEQC (bottom) patient data sets were plotted in patients with disease stage (st) 1, 2, 3, 4, and 4S, respectively, with patient numbers shown in parentheses (* *p* < 0.05).

These results strongly suggested that high expression of TPX2 was related to augmented risk of relapse and death. In two NB patient groups belonging to all tumor stages, high *TPX2* gene expression was associated with decreased relapse-free, and event-free survival rates (Figure 7A). It should be noted that *TPX2* gene expression was significantly higher in patients with stage four disease compared to stages 1, 2, 3, and 4S (Figure 7B).

**Figure 8:**
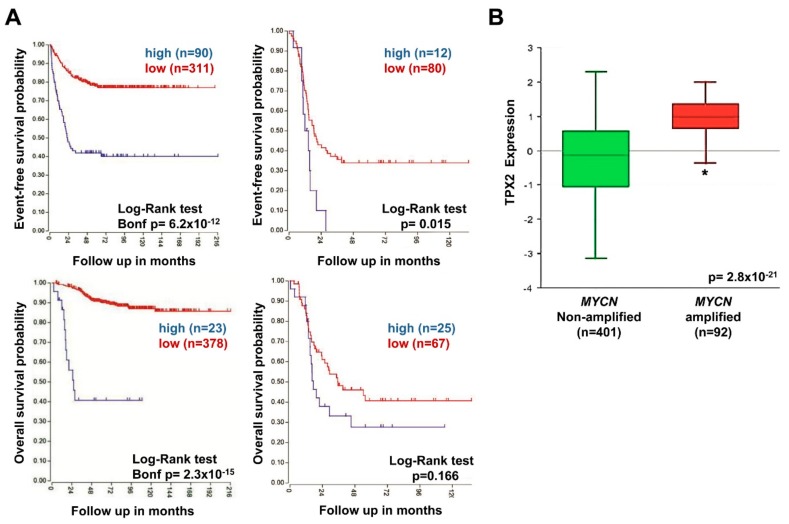
Neuroblastoma patient outcomes based on *TPX2* expression and *MYCN* amplification. (**A**) Using the neuroblastoma SEQC patient data-set in the R2 Genomics Analysis and Visualization Platform (http://r2.amc.nl), patients were divided into high (blue) and low (red) *TPX2* gene expression groups by median-centered Log2 ratios and survival curves were generated for patients with *MYCN* non-amplified tumors (left) and with *MYCN* amplified tumors (right). Event-free survival (top) and overall survival (bottom) curves are shown, with patient numbers in parentheses. (**B**) Relative *TPX2* expression levels were plotted in patients with *MYCN* non-amplified and amplified tumors (* *p* < 0.05).

To evaluate the possible association between *TPX2* expression and the outcome of patients with tumors carrying *MYCN* amplification and tumors with *MYCN* single copy, the two NB patient groups were stratified by the presence or lack of amplified *MYCN*. Patients with high *TPX2* gene expression had reduced event-free and overall survival in groups with both *MYCN* amplified and non-amplified tumors (Figure 8A), but these trends did not reach statistical significance. Furthermore, *TPX2* gene expression was also significantly higher in patients with *MYCN* amplified tumors compared to *MYCN* single copy tumors (Figure 8B). Our results demonstrate the association of augmented *TPX2* gene expression with poor outcomes in all NB patients’ subsets.

**Figure 9:**
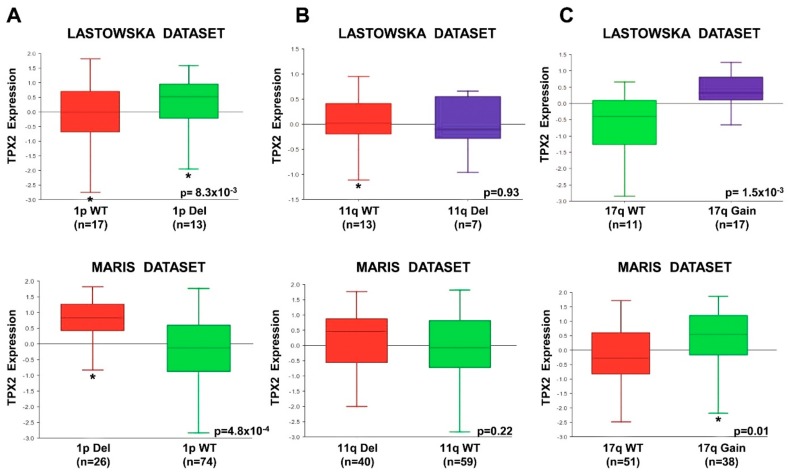
*TPX2* Expression Compared to Chromosome 1p, 11q, and 17q status. Using the R2 Genomics Analysis and Visualization Platform, *TPX2* gene expression in the Lastowska (upper) and Maris (lower) data sets was compared in patients with wild-type chromosome 1p or with 1p deletion (**A**), in patients with wild-type chromosome 11q or with 11q deletion (**B**), and in patients with wild-type chromosome 17q or with 17q gain (**C**). Patients’ numbers are shown in parentheses. (WT = Wild Type; Del = Deletion) (* *p* < 0.05).

In order to establish whether *TPX2* gene expression was correlated to chromosome 1p deletion, 11q deletion, and 17q gain, abnormalities associated with poor NB patient outcomes [54], we analyzed the results from datasets using the R2 Genomics Analysis and Visualization Platform. In two independent datasets, *TPX2* gene expression was significantly higher in the tumors from patients with chromosome 1p deletion, and 17q gain compared to those with normal chromosome (Figure 9). There was no difference in *TPX2* gene expression with respect to 11q deletion or a normal chromosome (Figure 9).

Moreover, the patients of our dataset with augmented *TPX2* expression had very poor outcomes, because all died of disease (Table 1).

## 3. Discussion

*GOLPH3* is an oncogene often amplified and over-expressed in many human tumors, and its increased expression anticipates poor patient prognosis [29,30,31,32,33,34]. *GOLPH3* is the first oncogene found involved in regulating Golgi secretory functions. It has been demonstrated that DNA damage triggers dramatic reorganization of the Golgi shape, resulting in its dispersal throughout the cytoplasm via Golgi protein GOLPH3 [23]. Because little was known regarding the effects of DNA damage on the Golgi, and about the Golgi shape in neuroblastic tumors, we focused on the underlying molecular mechanism of GOPLH3 in NB cells.

To investigate the mechanism of resistance of NB cells to elimination induced by DNA damaging agents, we used two inducible NB cell models: IMR-32 cell line with amplification of *MYCN* oncogene, and SH-SY5Y cell line with *MYCN* single copy but with high level of c-MYC expression. *MYCN* oncogene is associated with aggressive disease course and poor outcome of NB patients [1,2,3,4,5], and c-MYC is activated as a potent oncogene in a well-defined subset of high-risk NB cases too [55]. The clinical outcome of NB patients with high level of c-MYC expression is almost identical to that of NB patients with *MYCN* amplification [56].

DNA damage is well known to drive the activation of DNA repair, cell cycle arrest, and transcriptional changes [37,57,58]. We induced DNA damage in IMR-32 and SH-SY5Y human NB cell lines with curcumin, a polyphenol extracted from a tropical plant native to South and Southeast Asia that has been shown to have anti-inflammatory, anti-viral, and anticancer activities [59,60]. It has been demonstrated that curcumin enhanced the therapeutic efficacy of traditional chemotherapeutic drugs, cisplatin, doxorubicin, 5-fluorouracil, and gemcitabine, however, under some circumstances its effects can be contradictory [61]. Curcumin has been reported to demonstrate a good anti-proliferative activity on tumor cells by inducing apoptosis [62] in NB cells. DNA damage induced by topoisomerase II-mediated DNA cleavage is the mechanism through which curcumin induces apoptosis [63]. It is to be noted that in vitro studies have given direct evidence that curcumin induced G2/M arrest and DNA damage in tumor cells [64,65,66,67,68,69,70,71,72], but it acts as a protective agent for normal cells [73]. Moreover, curcumin modulates autophagy in NB cells [74]: the role of autophagy in NB is an emerging field of great interest in both biological and therapeutic aspects [75,76,77,78].

We found that GOLPH3 was over-expressed in NB cell lines after DNA damage caused by curcumin, and in primary NB human tissue sections from surgical resection post-chemotherapy treatment inducing DNA damage. Moreover, curcumin treatment reduced the activation of MAP-kinases, induces apoptosis, autophagy, and GOLPH3 fragmentation in NB cells. Furthermore, our results showed that DNA damage drastically induced Golgi dispersal throughout the cytoplasm in NB cells. DNA damage induces Golgi fragmentation in several human cell lines (HeLa, HEK293, NRK, MCF7, and MDA-MB-231), in primary human endothelial cells of the umbilical vein, as well as in primary mouse embryonic fibroblasts, and in mouse hepatocytes [23]. Thus, Golgi fragmentation is a common feature of DNA damage response in mammalian cells. Normally, the GOLPH3 pathway regulates the tensile forces upon Golgi complex, contributing to its right organization around the nucleus. Since this pathway appears conserved in many different species, determining Golgi shape could be just a side effect role of GOLPH3 dependent trafficking from Golgi to plasma membrane. We demonstrated that the Golgi fragmentation and dispersal throughout the cytoplasm is functionally important for NB cell survival after DNA damage. The Golgi dispersal is the demonstration that the GOLPH3 pathway is activated in response to DNA damage, with consequences for trafficking function, as well as for NB cell survival. Over-expression of GOLPH3 with Golgi fragmentation caused by DNA damage induced by curcumin seems to confer a survival advantage to NB cells. We have observed that cells drastically stop growing or die when incubated with curcumin for 48 h, but, if they survive, they rapidly recover after curcumin depletion, starting to grow again very rapidly, in spite of so detrimental a treatment. The GOLPH3 activation, together with Golgi dispersal throughout the cytoplasm, were co-localized with autophagy markers expression, that could be the process through which these cells better resist and survive. Over-expression of GOLPH3, by the way, is reported to promote proliferation and tumorigenicity in many other kinds of human cancer [29,30,31,32,33,34]. Changes in the Golgi to plasma membrane trafficking that happen upon Golgi fragmentation following DNA damage may be important for the enhanced survival benefit supporting this pathway, although the crucial resultants have not been identified yet in NB. The DNA damage response is a well-known driver of cancer progression [37]. In addition, the resistance consequent to the recovery from DNA damage given by GOLPH3 over-expression has specific relevance to tumor cells, as most standard therapeutic procedures include DNA damaging agents. Therefore, our findings support the use of GOLPH3 expression levels as a clinical marker to predict responsiveness to DNA damaging cancer therapies of NB patients. Whether GOLPH3 regulates chemo-resistance in NB needs to be investigated in further studies. Furthermore, novel molecules able to interfere with GOLPH3 pathway may produce some benefits when used in combination with standard DNA damage therapeutic factors.

The role of RAS/MAPK pathway signaling in NB tumor cells is poorly understood. Activating mutations in the genes components of RAS/MAPK pathway have been identified in a small group of NB tumors at diagnosis [79] and more frequently at relapse [80]. In addition, the expression of the gene for the Ras GTPase-activating protein (RasGAP) NF1 is also related with the NB patient outcome, suggesting a significant role for RAS/MAPK pathway in NB pathogenesis and disease relapse [81,82]. Patients with high-risk NB have very poor outcomes, and an extensive comprehension of the pathways involved in NB pathogenesis will help in the development of improved therapies. In this study, we demonstrate that DNA damage augmented the expression of TPX2, the molecule that controls microtubule nucleation around centrosome, where Golgi is normally clustered. A relation between Golgi and TPX2 protein has been described, according to which Golgi regulates mitotic spindle formation by promoting the activation of the spindle assembly factor TPX2 [83]. Our results demonstrate a connection between TPX2 protein related to DNA damage, and GOLPH3 that enhances cell survival in response to DNA damage in NB cells. Our experiments showed that the over-expression of MYCN and c-MYC proteins independently correlate with an augmented TPX2 expression. It has been described *TPX2* as a co-regulator of *MYC* pathway too [84]. As TPX2 expression is associated with NB cell proliferation and NB patient outcome and prognostic features, we suggest a role for TPX2 as a novel oncoprotein in NB. Although we have demonstrated the association between TPX2 and a number of critical NB prognostic variables, other studies are indispensable to determine whether TPX2 expression is independently associated with NB patient outcomes. We have shown that NB cases with 1p deletion and 17q gain have higher TPX2 levels, suggesting that increased *TPX2* gene expression may be the cause of the poor prognosis for this group of patients. Further studies are ongoing to identify whether other genetic or epigenetic events are responsible for regulation of *TPX2* gene expression.

**Figure 10:**
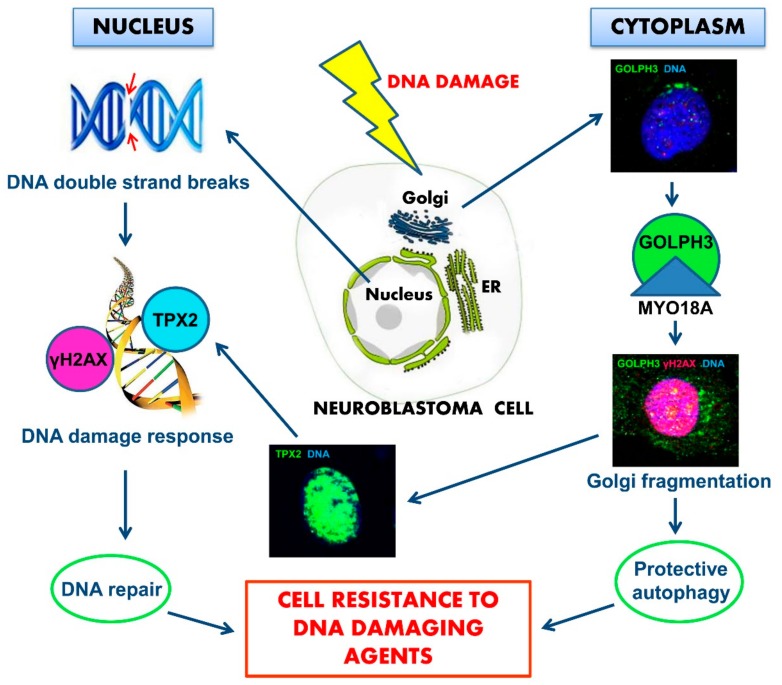
Schematic model of the proposed neuroblastoma (NB) cells resistance to death induced by DNA damaging agents. DNA damage agents induce chromosomal double-strand breaks in the nucleus, where chromatin is physically connected to the nuclear envelope, which is continuous with endoplasmic reticulum (ER). Membrane areas of close apposition connect ER with Golgi apparatus and with the plasma membrane. DNA double-strand breaks generate a signal leading to augmented Golgi protein GOLPH3 interaction with MYO18A, inducing Golgi dispersal and impaired intracellular trafficking with functional consequences for the cell. In the cytoplasm, Golgi fragmentation is accompanied by activation of protective autophagy. In the nucleus, over-expression of TPX2 induces DNA damage response with resulting DNA repair. The combined effects of the two intertwined mechanisms can support enhanced survival and NB cell resistance to chemotherapy.

Definitely, we propose that in response to DNA damage, GOLPH3 is activated and its interaction with MYO18A is reinforced, leading to an increased tensile force on the Golgi and consequently to Golgi fragmentation and dispersal in the cytoplasm of NB cells. Then, while in the nucleus, the activation of TPX2 induces the DNA damage response and, in the cytoplasm, Golgi dispersal leads to altered cell trafficking. Moreover, Golgi fragmentation is accompanied by activation of protective autophagy so NB cell can repair DNA. Over-expression of GOLPH3 and TPX2 confers resistance to killing by DNA damaging agents and enhances survival of NB cells (Figure 10). This mechanism suggests physical continuity between DNA in the nucleus and Golgi in the cytoplasm. The upstream mechanism leading to the activation of the two intertwined processes that cause Golgi dispersal altered trafficking, and DNA repair in response to DNA damage, is yet to be determined.

## 4. Materials and Methods

### 4.1. Cell Cultures

Human certified NB cell lines IMR-32 and SH-SY5Y were obtained from ICLC-Interlab Cell Line Collection (San Martino-IST, Genova, Italy), while HEK-293 cell line was obtained from The Global Bioresource Center (ATCC, Manassas, VA, USA). HEK-293 cells, chosen as non-tumorigenic control cells because they show a neuronal lineage phenotype and express adrenergic markers [85], were utilized at low culturing passage, to avoid any possible tumorigenic properties [86]. Cells were cultured in DMEM High Glucose (EuroClone, Milano, Italy) supplemented with 10% FBS (Gibco, Waltham, MA, USA) and maintained at 37 °C in a humidified atmosphere of 95% air and 5% CO_2_. The genomic identity of each line was regularly established by array-CGH, and cell lines were always tested to certify lack of mycoplasma contamination.

### 4.2. Curcumin and Cells Growth

Curcumin (diferuloylmethane) [87] was purchased from Alfa Aesar (GmbH & Co KG, Karlsruhe, Germany), with a purity of 95%, dissolved in dimethyl sulfoxide (DMSO) at a final concentration of 100 mM and stored at −20 °C until used. For in vitro experiments, curcumin was diluted before use in complete medium to contain <0.1% DMSO. NB cell lines were treated with a series of concentrations of curcumin from 2 to 80 μM for 24 h at 37 °C to evaluate the effects on cell growth and survival in a dose dependent manner. The curcumin concentrations that gave a significant effect with low cytotoxicity were tested on 24 and 48 h treatment experiments. After 48 h of treatment with the most significant curcumin concentration, medium was changed with normal fresh DMEM and cells were counted after 1, 2 and 3 days to observe their recovery degree. At each harvest point, viable cells were trypsinized and counted in Trypan blue. Control cells were cultured in the same DMSO concentration (0.1%).

### 4.3. Western Blot Analysis

Cells were lysed in Staph-A buffer (1.6 mM NaH_2_PO_4_; 8.6 mM Na_2_HPO_4_; 1% Triton X-100; 0.1% SDS; 0.1% NaCl; 0.5% NaDoc; 2 mM AEBSF; 20 mg/mL each of aprotinin and leupeptin) and the obtained protein lysates (100 µg each) were subjected to SDS-PAGE electrophoresis and transferred to PVDF membrane (Millipore, Burlington, MA, USA). Blots were probed with the following monoclonal antibodies: anti-PARP (Abcam, Cambridge, MA, UK), anti-phospho-Histone γH2AX (Millipore), anti-TPX2 (Santa Cruz Biotechnology, Dallas, TX, USA), and anti-c-MYC (Santa Cruz Biotechnology); and polyclonal antibodies: anti-GOLPH3 (Abcam), anti-Beclin-1 (Novus Biologicals, Littleton, CO, USA), anti-LC3A (Cell Signaling Technology Inc., Danvers, MA, USA), anti-MYO18A (Abnova, Taipei, Taiwan), anti-Histone H2AX (Sigma, St Louis, MO, USA), and anti-MYCN (Novus Biologicals). Proteins were visualized by West Dura Extended chemiluminescent detection (Thermo Scientific, Carlsbad, CA, USA) using HRP-conjugated secondary antibodies (Thermo Scientific). Blots were re-probed with anti-β-actin (Santa Cruz Biotechnology) as loading control. Bands signal intensity was measured by densitometry using Image Lab 6.0 software (ChemiDoc, Bio-Rad, Hercules, CA, USA), and normalized to the loading control.

### 4.4. Kinase Activation Assay

Cells were starved 18 h and then lysed in order to assess the activated p38, ERK1/2, and Akt levels. Cell lysates were subjected to SDS-PAGE and transferred to PVDF membrane. The blots were then probed with a phospho-specific monoclonal antibody against P-p38 and with polyclonal antibodies against P-ERK1/2 and P-Akt (Cell Signaling Technology). To check the total amount of proteins loaded, the blots were re-probed with polyclonal antibodies against p38, ERK2 (Santa Cruz Biotechnology), and Akt (Cell Signaling Technology). Band signal intensity was measured by densitometry and normalized to each total protein expression.

### 4.5. Patients and Tumor Samples

The primary NB human tissue sections analyzed included three from pre-chemotherapy (NB1, NB2, NB3) and 10 from post-chemotherapy surgical resection (NB1 to NB10) (Table 1), all diagnosed by the Italian Association of Pediatric Hematology and Oncology (AIEOP). The post-chemotherapy surgical resections were obtained after induction with rapid COJEC. Rapid COJEC (two courses of carboplatin, etoposide, vincristine; four courses of cisplatin, vincristine; two courses of etoposide, cyclophosphamide) is a time-intensive chemotherapy regimen administered at 10-day intervals. These patients harbored chemotherapy resistant tumors. All surgical resections were taken from high-risk NB patients, as defined by the International Neuroblastoma Risk Group (INRG) classification system [88], enrolled in the HR-NBL-1 protocol (SIOPEN) [89]. Informed consent was obtained from all individual participants included in the study. This study was approved by the Institutional Ethics Committee of Regione Liguria (Prot. n° IGG-NCA-AP-2016; 12/15/2016).

### 4.6. Immunofluorescence Analysis

Immunofluorescence analysis was performed on formalin-fixed, paraffin-embedded NB specimens (4 μm-thick) or on cytospins as previously described [90]. The following antibodies were used: monoclonal anti-phospho-Histone γH2AX (1:20, Millipore) and anti-TPX2 (1:20, Santa Cruz Biotechnology); polyclonal anti-GOLPH3 (1:10, Abcam), and anti-Beclin-1 (1:30 Novus Biologicals). We used isotype matched non-binding mAbs in all antibody staining experiments to avoid nonspecific reactivity. Results were photographically documented using fluorescence microscope Axio Imager M2 equipped with ApoTome System (Carl Zeiss, Oberkoche, Germany). For cytospins, values represent percentages from at least 1000 counted positive and negative cells. For NB specimens, each tumor area tested contained malignant cells as assessed by histologic examination. Quantification of immunofluorescence positive tumor cells was performed on serial tissue sections, thus allowing quantification in tumor areas selected by the pathologist. Tumor cells were identified in each sample using the NB specific marker NCAM (NB56) [91]. The proportion of immunofluorescence positive cells counted was at least 100 to 1000 cells and reported in the percentage for the subsequent statistical analysis.

### 4.7. Detection of Apoptotic Cells

Apoptotic cells were detected using in situ Cell Death Detection Kit (Roche, Basel, Switzerland) as described previously [92]. The elements considered to estimate the apoptosis degree were chromatin condensation, the presence of cytoplasmic fragments (apoptotic bodies), and of intra- and extracellular chromatin fragments (micronuclei). Values represent percentages from at least 1000 counted apoptotic and non-apoptotic cells.

### 4.8. Genomic Profile Determination

We analyzed by array-CGH the genome wide copy number variations in the DNA extracted from the 10 NB samples here considered coming from pre-chemotherapy surgical resection using Human Genome CGH Microarray 4X 180K Kit (Agilent Technologies, Santa Clara, CA, USA), with a mean resolution of approximately 25 kb, as previously described [93]. Tumor DNAs were extracted using the QIAamp DNA Extraction Kit (Qiagen, Hilden, Germany), according to the manufacturer’s instructions. Images of the array were acquired with the Agilent C Scanner (Agilent Technologies), which were processed using the Agilent Feature Extraction 10.5 Software. The data were analyzed using the Genomic Workbench 7.0.40 software (Agilent), the altered chromosomal regions and breakpoints events were detected using the algorithm ADM-1 (threshold 10) with 0.5 Mb window size to reduce false positives [93]. The raw data are stored in the BIT-NB Bio Bank of IRCCS Gaslini.

### 4.9. TPX2 Gene Expression Analysis

We evaluated the association of *TPX2* gene expression with NB patients outcomes, using public microarray data from two different NB patients datasets (SEQC-498 and Versteeg obtained from the R2 Genomic Analysis and Visualization Platform (http://r2.amc.nl) [94,95]. *TPX2* gene probe-sets (SEQC-498 probe-set NM_012112; Versteeg probe-set 210052) in each database with the highest average signals were selected for analysis. The best cut-off for survival analyses was determined as the expression value where the statistic log-rank for the separation of survival curves reached its higher level.

### 4.10. Statistical Analysis

The microarray data generated from the R2 Genomic Analysis and Visualization Platform was examined using the R2 program for analysis and visualization of microarray data (http://r2.amc.nl). We performed on-line Kaplan-Meier analyses and evaluation of *TPX2* expression comparing different patient subgroups for the two NB cohorts. Next, we downloaded the resulting survival curves, box plots, and p-values (obtained with log-rank test). The Mann-Whitney U test was used for the comparison of *TPX2* expression among the different NB patients’ subgroups.

All experiments were performed at least three times. For all analyses, the significance of differences between experimental samples and controls was determined by ANOVA analysis with Bonferroni’s Multiple comparison Test (* *p* < 0.05; ** *p* < 0.01; *** *p* < 0.001).

## 5. Conclusions

Our findings suggest that GOLPH3 expression level may represent a possible clinical marker of NB patient responsiveness to DNA damaging therapies. Novel molecules able to interfere with the GOLPH3 pathway may produce therapeutic benefits when combined with standard DNA damaging therapeutic factors in NB. TPX2 too can therefore represent a molecular target for the development of new drugs. The results of this study provide several starting points for future investigations as part of the preclinical and clinical fight against NB.

## Figures and Tables

**Table 1:** Clinicopathological characteristics of Neuroblastoma (NB) patients and post-chemotherapy tumor samples examined.

Case	Age Months	Stage INSS	Therapy Protocol	*MYCN* Status	Del 1p	Del 11q	Gain 17q	Chemotherapy (Rapid COJEC)	Immunotherapy (Anti-GD2)	Response to Chemotherapy	Relapse	Overall Survival
NB 1	66	3	HR-NBL-1	Amplified	Y	Y	Y	Y	N	Partial	Y	Dead of disease
NB 2	70	3	HR-NBL-1	Amplified	N	N	Y	Y	N	Partial	Y	Dead of disease
NB 3	178	3	HR-NBL-1	Amplified	N	Y	Y	Y	N	Partial	Y	Dead of disease
NB 4	80	3	HR-NBL-1	Amplified	Y	Y	Y	Y	N	Partial	Y	Dead of disease
NB 5	53	4	HR-NBL-1	Amplified	Y	N	Y	Y	N	Partial	Y	Dead of disease
NB 6	20	4	HR-NBL-1	Amplified	Y	N	Y	Y	Y	Partial	Y	Dead of disease
NB 7	37	3	HR-NBL-1	Amplified	Y	Y	Y	Y	N	Partial	Y	Dead of disease
NB 8	65	4	HR-NBL-1	Non-Amplified	N	N	Y	Y	Y	Partial	Y	Dead of disease
NB 9	63	4	HR-NBL-1	Non-Amplified	N	Y	Y	Y	N	Partial	N	Dead of disease
NB10	7	4	HR-NBL-1	Amplified	Y	N	Y	Y	N	Partial	Y	Dead of disease

INSS = International Neuroblastoma Staging System; Y = yes; N = no; Del = Deletion; 1p, 11q, 17q = chromosomes arms affected by deletion or gain in NB.

## Supplementary Materials

Supplementary materials can be found at https://www.mdpi.com/1422-0067/20/19/4764/s1.

## Abbreviations

NB	Neuroblastoma
*MYCN*	v-myc myelocytomatosis viral related oncogene, neuroblastoma derived
GOLPH3	Golgi phosphoprotein 3
MYO18A	Myosin XVIIIA
H2AX	H2A histone family, member X
TPX2	microtubule-associated, homolog (Xenopus laevis)
DNA-PK	DNA dependent protein kinase complex
MAPKs	mitogen-activated protein kinases
p38	p38 MAPK
ERK1/2	extracellular signal-regulated kinase 1 and 2
Akt	RAC-alpha serine-threonine-protein kinase
Ras	Rat Sarcoma family of GTP-ases
GAP	GTPase-activating protein
PARP	Poly ADP-Ribose Polymerase
LC3A	Microtubule Associated Protein 1 Light Chain 3 Alpha
ER	endoplasmic reticulum
TUNEL	Terminal deoxynucleotidyl transferase dUTP nick end labeling
CGH	Comparative Genomic Hybridization
INSS	International Neuroblastoma Staging System
INRG	International Neuroblastoma Risk Group
SIOPEN	International Society of Pediatric Oncology European Neuroblastoma
AIEOP	Italian Association of Pediatric Hematology and Oncology

## Appendix A

### Rapamycin, Bortezomib, and Colchicine Treatments

Rapamycin, Bortezomib and Colchicine were tested as DNA damage and Golgi fragmentation inducers for their known anti-proliferative and apoptotic effect on NB cells [50,51,52]. These compounds have been studied as possible drugs for NB therapy too [96,97,98], and, furthermore, all them have shown an involvement in autophagy induction, in NB or in other kind of human cancers [75,99,100]. Rapamycin was purchased from InvivoGen (San Diego, CA, USA), with a purity of more than 95%, and dissolved in DMSO at a final concentration of 10mM; Bortezomib was purchased from Cell Signaling Technology Inc. (Danvers, MA, USA), with a purity of 99%, and dissolved in DMSO at a final concentration of 1mM; Colchicine was purchased from InvivoGen with a purity of 95%, and dissolved in distilled water at a final concentration of 25mM. The drugs were divided into aliquots and stored at -20 °C until used for in vitro experiments, when they were diluted in complete medium. NB cell lines were treated with a series of drugs concentrations for 24 and 48 h at 37 °C to evaluate the dose that gave a significant effect with the lowest degree of cytotoxicity.
