# Supplementary material for: Role of GOLPH3 and TPX2 in Neuroblastoma DNA Damage Response and Cell Resistance to Chemotherapy
Source: Int J Mol Sci. 2019 Sep 25;20(19):4764. doi: 10.3390/ijms20194764 (PMC6801815; doi:10.3390/ijms20194764)
Supplement: Supplementary file 1 [file ijms-20-04764-s001.pdf]

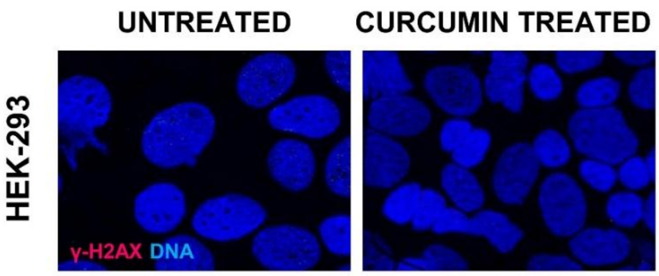

Supplementary Figure 1. Immunofluorescence analysis of non tumoral control HEK-293 cells cultured with 20 µM curcumin 48 hours using anti-γH2AX (red). Cells were counterstained with DAPI to visualize nuclei (blue). Untreated cells were cultured with 0.1% DMSO. (Magnification 40x).

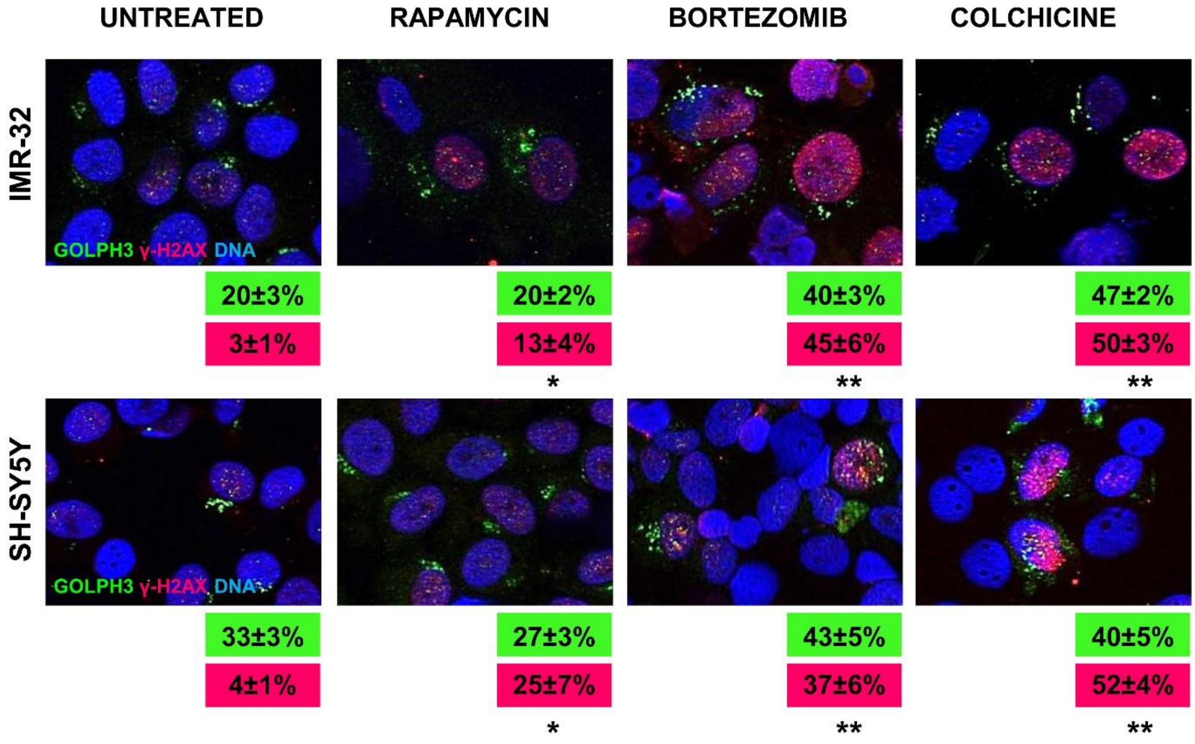

Supplementary Figure 2. Immunofluorescence analysis of IMR-32 and SH-SY5Y cells cultured respectively with 300 nM or 500 nM Rapamycin, with 2 nM or 3.5 nM Bortezomib and with 0.02 µM or 0.05 µM Colchicine for 48 hours using anti-GOLPH3 (green) and anti-γH2AX (red) and compared to untreated cells. Cells were counterstained with DAPI to visualize nuclei (blue). (Magnification 40x). In green and red boxes are reported the percentages of GOLPH3 and γH2AX positive cells respectively. Data are representative of three independent experiments ± S.D.
